# The Assessment of the Effect of Gaze Direction Instruction on the Stabilisation During Artistic Gymnastic Landing

**DOI:** 10.1002/ejsc.70137

**Published:** 2026-02-06

**Authors:** K. Pavlasová, L. Bizovská, L. Rupčík, R. Farana, M. Janura

**Affiliations:** ^1^ Department of Natural Sciences in Kinanthropology Faculty of Physical Culture Palacký University Olomouc Olomouc Czechia; ^2^ Department of Gymnastics Dance Fitness & Combat Sports Comenius University Bratislava Bratislava Slovakia; ^3^ Department of Human Movement Studies Faculty of Education University of Ostrava Ostrava Czechia

**Keywords:** biomechanics, musculoskeletal, performance

## Abstract

The aim of this study was to assess the effect of gaze direction instruction on postural stabilisation and muscle activity during landings after various motor tasks in artistic gymnastics. Eighteen female gymnasts (aged 14.0 ± 2.7 years) performed four different landing tasks: drop landing, backward somersault, forward somersault (SF) and a backward acrobatic series (AS). Gaze was directed either downward or straight ahead during landings. Muscle activity was recorded from six lower limb muscles and postural stabilisation was assessed using dynamic stability indices and time to stabilisation. Results showed a significant effect of motor task on muscle activity and stabilisation, with AS and SF presenting higher stabilisation demands. Gaze direction instruction had limited influence on muscle activation patterns and stabilisation, with limited manifestation in dynamic stability indices. Interactions between gaze and motor task were significant only for sagittal dynamic stability in longer time intervals. These findings suggest that motor task complexity primarily governs landing stabilisation, whereas gaze direction plays a minor role in lower limb muscle activity.

## Highlights

- The type of motor task performed before landing significantly affected muscle activity and landing stabilisation.
- The acrobatic series and forward somersault exhibited the highest stabilisation demands.
- Interactions between gaze direction and motor task occurred primarily in the sagittal plane.

## 1 Introduction

Acrobatic elements of the somersault type are among the basic skills of artistic gymnasts (George 2010). Their execution requires a high degree of spatial orientation (Davlin et al. 2001a, 2001b; Heinen 2015), which includes information from the visual, vestibular and somatosensory systems (Asseman et al. 2005; Davlin et al. 2002). Due to the mechanism of the flip elements, the systems involved in spatial orientation are stressed beyond their optimal functional range (Davlin et al. 2002; Bardy and Laurent 1998). In the flight phase, otoliths in the vestibular system and mechanoreceptors located in tissues are outside this functional range and cannot provide accurate information about the change in body orientation during the execution of the flip elements. In this case, vision is considered the main source of information (Bardy and Laurent 1998; Davlin et al. 2001b). This is also true for its use during the landing, when it plays an important role to ensure effective stabilisation (Davlin et al. 2002; Gaerlan et al. 2012).

The manipulation of visual feedback during all or part of an acrobatic element has been reported in several studies involving the landing after a backward somersault (Davlin et al. 2001a, 2001b; Luis and Tremblay 2008). The lack of visual feedback, that is, performing a somersault without the possibility of visual inspection, resulted in a reduction in the number of successful landings evaluated according to the gymnastics' rules (Bardy and Laurent 1998). Although Davlin's studies (Davlin et al. 2001a, 2001b) did not find a statistically significant effect of limited or absent visual feedback, the authors observed a consistent trend of reduced body control and stability during landing when visual feedback was limited.

As information from the visual system is used to orient during flight phase, some authors have suggested that gaze direction alone could also affect movement control during the flight phase and the landing. Heinen et al. (2012) demonstrated that changing the position of the light signal marking the point on which the participants were asked to fix their gaze on a landing mat affected participants' movements. Specifically, when participants fixed their gaze on different places during a backward somersault dismount from the bars, this change influenced the lower body kinematics during the flight phase and landing location. Similarly, more recent studies conducted on a trampoline using eye‐tracking technology showed that gymnasts find a fixation point on the trampoline during the second phase of the backward somersault, which they look at throughout the entire landing (Natrup et al. 2020), and that the existence of a fixation point is extremely important when landing after more challenging types of somersaults with twists (Natrup et al. 2021).

However, not only gaze direction or the presence of a fixation point but also the onset of gaze fixation has been a subject of research interest. For example, during the execution of a straight jump with a 360° turn, gymnasts begin to fix their gaze as early as 110 milliseconds prior to landing (Sato et al. 2016). The timing of gaze fixation onset is associated with the athlete's skill level, with more experienced athletes exhibiting a later onset compared to their less skilled counterparts (Natrup et al. 2020, 2021). The importance of fixation onset lies in the body's preparation for landing through the preactivation of appropriate muscle groups (Santello 2005). This mechanism ensures a successful and safe landing and is influenced by both the nature of the task preceding the landing (McNitt‐Gray et al. 2001) and the overall task difficulty (Santello 2005).

In the aforementioned studies examining the interplay between gaze and landing stabilisation, landing performance was assessed by certified judges. To the best of the authors' knowledge, no objective assessment of landing stabilisation—in terms of centre of mass or centre of pressure movement—has been reported in gaze‐related research. In contrast, studies focusing primarily on stabilisation commonly employ quantitative measures, such as time to stabilisation or the dynamic stability index (Colby et al. 1999; Fransz et al., 2024; Wikstrom et al. 2005), to enable a precise and objective evaluation of landing performance.

Considering all the aforementioned points, the aim of this study was to examine the effect of gaze direction instruction on landing stabilisation and muscle activity during landings performed after various motor tasks. It was hypothesised that altering the location of gaze fixation would lead to modifications in muscle activity associated with the preactivation of lower limb muscle groups. Furthermore, it was expected that these changes would be reflected in changes in stabilisation index values.

## 2 Materials and Methods

### 2.1 Participants

The research sample consisted of 18 female artistic gymnasts (age: 14.0 ± 2.7 years, height: 154.2 ± 8.5 cm and mass: 42.9 ± 9.4 kg) competing in the cadet, junior and senior categories. The gymnasts selected had completed a minimum of 4 years of specialised artistic gymnastics training, with training sessions of at least 15 h per week. Only gymnasts who were active competitors in national and international competitions, and who had not experienced any musculoskeletal injuries in the past 6 months, were included.

Participation was voluntary. All participants and their legal guardians were informed about the study's purpose, the measurement procedures, potential risks, injury prevention strategies, data handling methods and the option to withdraw from the research at any time. Gymnasts over the age of 18, or the legal guardians of younger participants, signed an informed consent form. The testing was conducted following approval from the Institutional Ethics Committee (no. 114/2023) and in accordance with the principles of the Declaration of Helsinki.

### 2.2 Data Collection

Testing took place on certified floor equipment (Diony/Spieth) in specialized gymnastics facilities equipped in accordance with Fédération Internationale de Gymnastique (FIG) in the Czech and Slovak Republic. Following an introduction to the testing protocol, gymnasts completed a self‐guided 10‐min warm‐up based on their personal training routines. Following, three tests were conducted to determine lower limb dominance: a ball kick, a step onto a platform and a balance recovery task. The limb that was identified as active in at least two of these tests was designated as the dominant lower limb.

Six sensors (Delsys Inc., Natick, MA, USA, sampling rates 1926 and 1111 Hz, based on the version and type of sensor used) were placed on the dominant lower limb to record muscle activity of the following muscles: *m*. rectus femoris, *m*. vastus medialis, *m*. biceps femoris, *m*. tibialis anterior, *m*. peroneus longus and *m*. gastrocnemius medialis. Muscles were identified by palpation by a skilled physiotherapist, and sensors were attached using special adhesive strips (Delsys Inc., Natick, MA, USA) and secured with kinesiology tape to prevent movement or falling off during rotations. These sensors were placed according to European recommendations for surface electromyography (SENIAM). An inertial sensor (Delsys Inc., Natick, MA, USA, sampling rate of 370.4 Hz) was secured on the gymnasts' back near the assumed position of the centre of mass at the level of the fourth lumbar vertebra to assess postural stabilisation during landing. All sensors (EMG and inertial sensor) were synchronised within a single Trigno base station.

The testing procedure was divided into 4 blocks according to the type of skill or acrobatic series preceding the landing: bipedal drop landing from a box with height of 30 cm (DL), a tucked somersault backward from the same box (SB), a tucked somersault forward preceded by a maximum of three running steps (SF), a backward acrobatic series consisted of a round‐off, back handspring and a tucked somersault backward (AS). During AS, participants could choose not to perform back handspring depending on their usual practise and preference. This was due to high variability of execution technique in recent years, with some gymnasts skipping the back handspring for various practical reasons. These motor tasks were selected because they are the most important basic skills for gymnasts.

Before each element or series, gymnasts were instructed on where to focus their gaze during the landing, either diagonally downward on the floor or straight ahead in front of them. The gaze direction was randomly assigned for each trial. The direction of gaze was controlled from the front by one person. In the case of imperfect gaze focus, the attempt was not considered valid. Two to three successful landings were recorded under each condition. A successful landing was defined as one with a maximum deduction of 0.3 points according to the International Code of Points for Women's Artistic Gymnastics (Fédération Internationale De Gymnastique 2022), accounting for minimal leg or torso separation or flexion. Landings with additional steps or jumps were not considered successful. The evaluation was done by one person who is a coach and judge with many years of experience.

### 2.3 Data Processing

#### 2.3.1 Muscle Activity

Due to discrepancy of sampling rates between used sensors, resampling procedure was performed as a first step of the analysis. The data were resampled to the unified 1000 Hz, band‐pass filtered using the 4^th^ order bidirectional Butterworth filter within a range of 30–350 Hz and full‐wave rectified. Muscle activity patterns were observed during the time interval of 300 ms before and 300 ms after contact time. Before proceeding, the quality of the signals was checked and signals reaching amplitudes above 3 mV or containing artefacts were excluded from the analysis. Following, all signals were normalised on the maximal amplitude observed during AS, individually for each participant regardless of gaze direction. Finally, integration within 20 ms time windows was performed (e.g., McNitt‐Gray et al. 2001).

#### 2.3.2 Stabilisation

Contact time, defined as the moment of contact between the feet and floor, was identified based on the local peak vertical acceleration following the last flight phase. Lower back accelerations in in the medial–lateral, anterior–posterior and vertical directions; and angular velocities in the sagittal, frontal and transverse planes were considered for further analysis within two time intervals—3 s following after contact time and 1 s following after contact time. The 3 s time interval was used as a standard time interval in which stabilisation indexes are usually computed (Wikstrom et al. 2005), 1s time interval was included as a more functional time for this type of sport, since 3 s of standing still could normally be considered as delaying the floor performance resulting in additional penalisation (Fédération Internationale De Gymnastique 2022). All accelerations and angular velocities were low‐pass filtered using a 4th‐order bidirectional Butterworth filter with a cut‐off frequency of 30 Hz. Time to stabilisation and dynamic stability index were computed after subtracting the mean value from the last 0.5 s (for the 3‐s interval) or 0.25 s (for the 1‐s interval) to establish a “zero” baseline. The dynamic stability index was calculated according to Wikstrom et al. (2005), time to stabilisation followed the method described by Colby et al. (1999) and assessed by Fransz et al. (2004).

#### 2.3.3 Statistical Analysis

All above‐written procedures of signal processing and computation of stabilisation indexes were performed based on the custom‐written MATLAB (MathWorks Inc. Natick, MA, USA) scripts. Integrated normalised muscle activity patterns were subjected to the 2‐way repeated measures Analysis of variance (ANOVA) using the statistical parametric mapping approach with MATLAB routines available on spm1d.org (Pataky 2012) to compare whole muscle patterns during prelanding and landing. Gaze direction (downward and straight ahead) and motor task (DL, SB, SF and AS) were used as within‐subject factors for comparison. Stabilisation indexes were compared using the 2‐way repeated measures ANOVA with the same factor set‐up in SPSS software (IBM Corporation, New York, NY, USA) on the significance level of 0.05. Bonferroni post hoc test was used for pair‐wise comparisons in cases the main effects or interaction were statistically significant. Normal data distribution was confirmed by the Kolmogorov–Smirnov test.

## 3 Results

### 3.1 Muscle Activity

**FIGURE 1:**
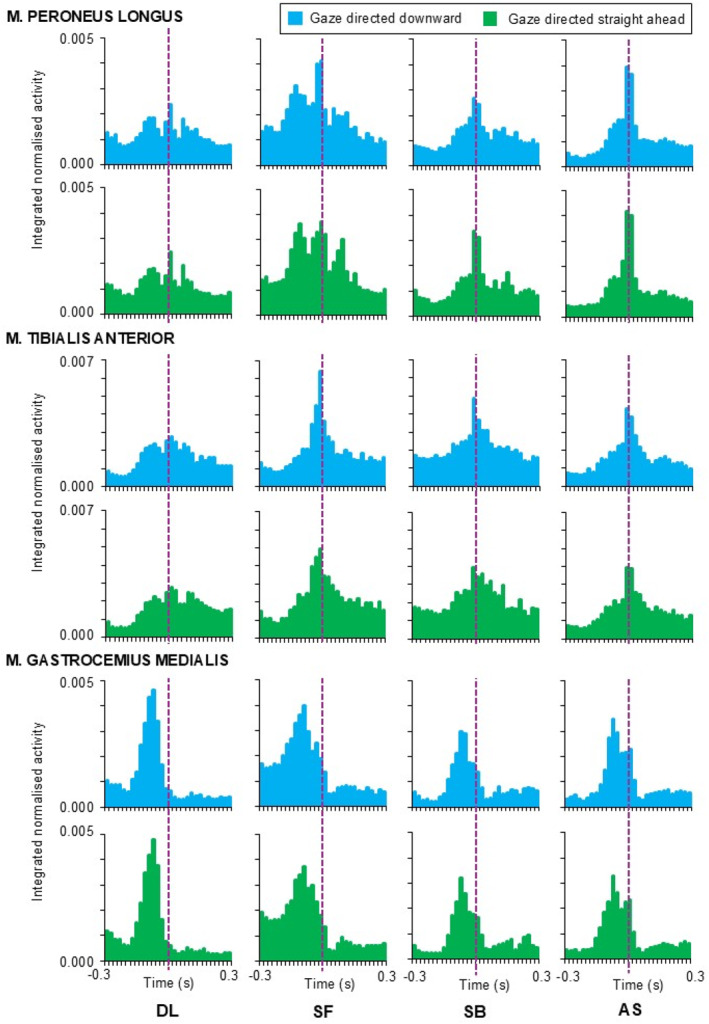
Mean muscle activity patterns observed for shank muscles during drop landing (DL), landing from a tucked somersault forward (SF), a tucked somersault backward (SB) and an acrobatic series (AS). Violet dashed line indicates the contact time.

**FIGURE 2:**
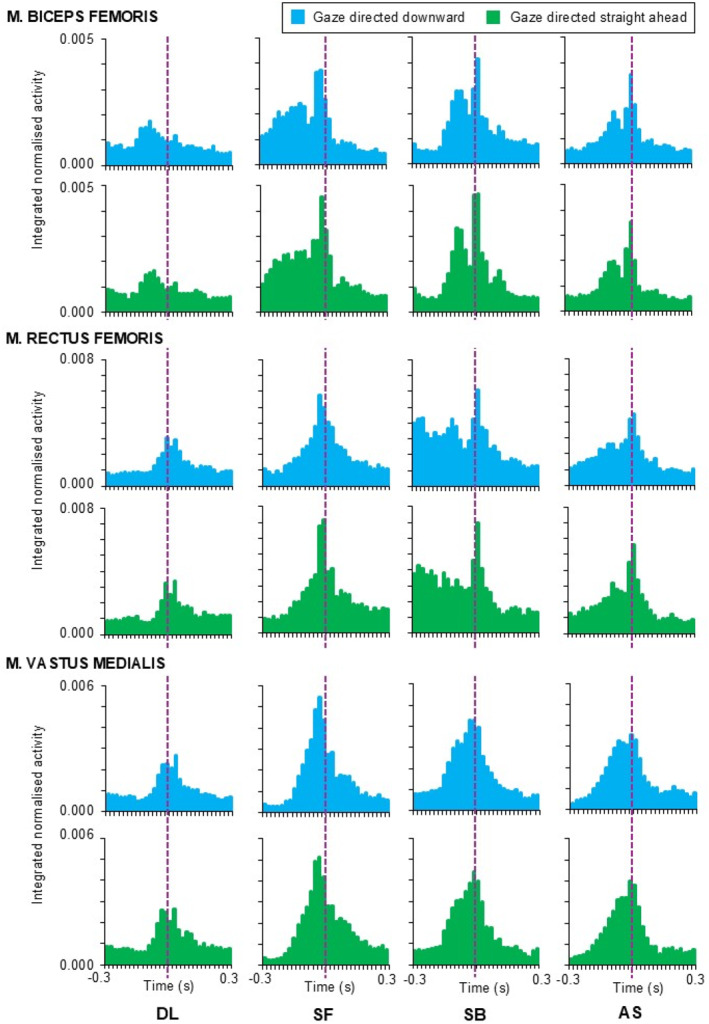
Mean muscle activity patterns observed for thigh muscles during drop landing (DL), landing from a tucked somersault forward (SF), a tucked somersault backward (SB) and an acrobatic series (AS). Violet dashed line indicates the contact time.

**FIGURE 3:**
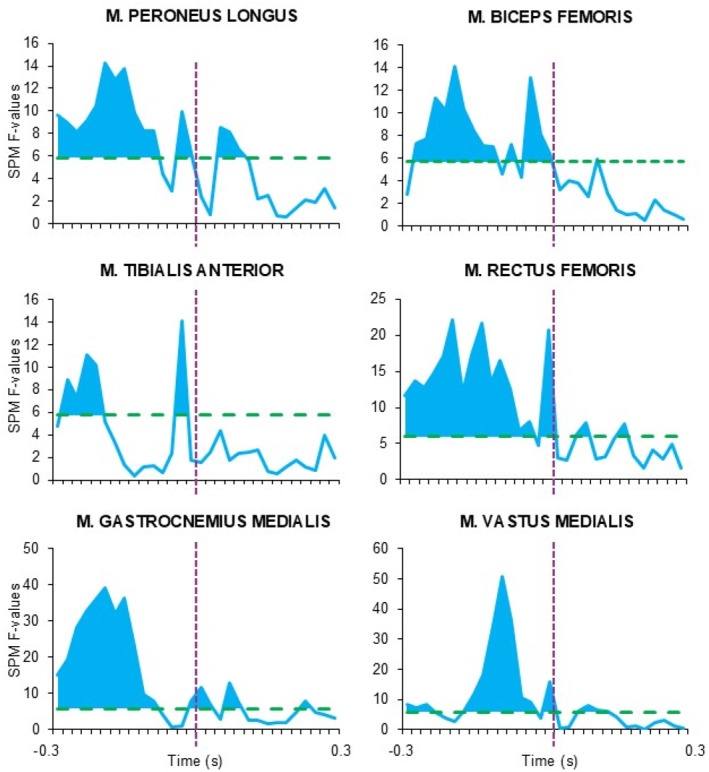
Statistical parametric mapping results for the effect of motor task with a green dashed line indicate critical F‐values, and blue areas above critical value indicating significant effect on a level of 0.05. Violet dashed line indicates the contact time.

Statistical parametric mapping analysis did not show the effect of gaze direction on any muscle activity patterns (Figures 1 and 2); however, the effect of motor task was apparent for all muscles during prelanding and for *m*. rectus femoris, *m*. vastus medialis, *m*. peroneus longu and *m*. gastrocnemius medialis even after contact time (Figure 3). No significant interaction between gaze direction and motor task was observed.

### 3.2 Stabilisation of Landing

**FIGURE 4:**
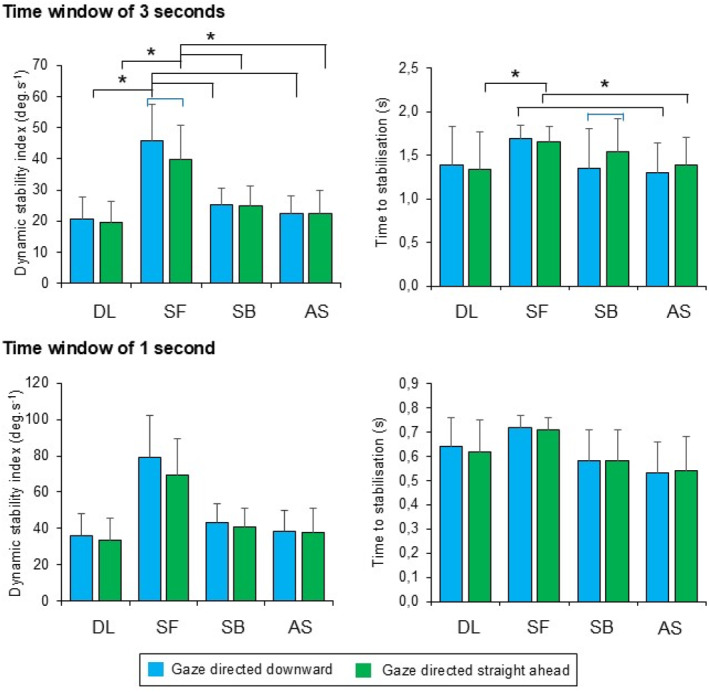
Descriptive results and results of pair‐wise comparisons for a significant interaction between motor task and gaze direction observed for sagittal dynamic stability index and time to sagittal stabilisation during drop landing (DL), landing from a tucked somersault forward (SF), a tucked somersault backward (SB) and an acrobatic series (AS). Black braces indicating significant effect of task, blue braces indicate significant effect of gaze direction.

Descriptive data are depicted in Figure 4. Due to the complexity of results, complete statistical results for stabilisation indexes can be found in the Supplementary file.

#### 3.2.1 Time Window of 3 s

The effect of motor task was statistically significant for dynamic stability indexes in all directions and planes (*p* ≤ 0.001). Based on the pair‐wise comparisons, sagittal dynamic stability index reached the highest value during landing from SF. Similar trends were observed for transverse and frontal dynamic stability index with reaching the lowest value during DL. Furthermore, transverse dynamic stability index was significantly higher during landing from AS compared to SB, and frontal dynamic stability index was significantly higher during landing from AS compared to SF. Medial–lateral, vertical and anterior–posterior dynamic stability indexes showed similar results after pairwise comparisons between conditions. All three indexes were significantly higher during landing after AS and SB compared to SF and DL; furthermore, medial–lateral dynamic stability index reached significantly higher value during landing from SF compared to DL, vertical dynamic stability index reached significantly higher value during landing from AS compared to SB.

Times to sagittal (*p* = 0.018), medial–lateral (*p* = 0.007) and vertical (*p* < 0.001) stabilisation were also significantly affected by motor task. Pairwise comparisons showed significantly higher time to sagittal stabilisation during landing from SF compared to AS. Times to medial–lateral and vertical stabilisation were higher during landing from DL compared to AS and SB, with time to vertical stabilisation being also significantly higher during DL compared to landing from SF.

Gaze direction straight ahead significantly worsened anterior–posterior dynamic stability index (*p* = 0.034) with a difference of 0.012 g between gaze conditions, transverse dynamic stability index (*p* = 0.025) and time to sagittal stabilisation (*p* = 0.050) with a difference of 0.050 s between gaze conditions. On the contrary, gaze direction downward significantly worsened sagittal dynamic stability index (*p* = 0.021), and time to vertical stabilisation (*p* = 0.040) with a difference of 0.042 s.

The interaction between motor task and gaze direction was observed to be significant for sagittal dynamic stability index and time to stabilisation (both *p* = 0.011 and Figure 4) with results indicating higher dynamic stability index with gaze directed downward during landing from SF (*p* = 0.003). Opposite was found for time to stabilisation during landing from SB (*p* = 0.007). Furthermore, results for dynamic stability index showed the highest value during landing from SF, regardless of gaze direction (all *p* < 0.001). Similar results were observed for time to stabilisation whilst gazing straight ahead when comparing landing from SF and AS (*p* = 0.046) and SF and DL (*p* = 0.046). With gaze directed downward, significantly higher values were also observed during SF compared to AS (*p* = 0.001).

#### 3.2.2 Time Window of 1 s

Effect of motor task was statistically significant (*p* < 0.05) for all dynamic stability indexes and times to stabilisation except from the time to frontal stabilisation (*p* = 0.065). The pair‐wise comparison showed that the highest value of sagittal dynamic stability index was observed during the landing after SF. For transverse and frontal dynamic stability indexes, similar results of pair‐wise comparisons were observed, showing lowest values reached during DL. Furthermore, significantly higher transverse dynamic stability index was observed whilst landing after AS compared to SB, and significantly higher frontal dynamic stability index was observed whilst landing after AS compared to SF. Anterior–posterior dynamic stability index was significantly higher whilst landing from AS compared to DL and SF. The same trend was observed for landing after SB, since it showed higher value compared to both DL and SF. For medial–lateral dynamic stability index, the lowest value was observed during DL, furthermore, significantly lower value was observed whilst landing from SF compared to both SB and AS. Vertical dynamic stability index reached highest values during landing from AS and a significantly lower value during DL as compared to SB.

When comparing time to sagittal stabilisation, results showed significantly higher value during landing after SF compared to both SB and AS. For time to transverse stabilisation, significantly lower value was observed during landing after AS as compared to SB and DL. Although the main effect of motor task was statistically significant for time to medial–lateral stabilisation (*p* = 0.047), pairwise comparison did not show any significantly different pairs. Time to vertical stabilisation reached the highest value during DL; however, the highest observed absolute difference between conditions was only of 0.091 s. Time to anterior–posterior stabilisation observed for landing after SB was significantly higher than after both SF and AS.

Gaze directed downward worsened sagittal (*p* = 0.031) dynamic stability index, however, improved the transverse (*p* = 0.028) dynamic stability index. Time to vertical stabilisation (*p* = 0.006) was also affected significantly by the gaze direction, however, the absolute difference of 0.013 s between gaze conditions does not seem practically relevant.

No significant interaction between motor task and gaze direction was found for 1s time window stabilisation data.

## 4 Discussion and Implications

The present study aimed to examine the effect of gaze direction instruction on postural stabilisation and muscle activity during landing after completing four motor tasks of varying difficulty. We hypothesized that gaze direction would differentially influence landing stabilisation and muscle activity patterns depending on the motor task performed. However, although we found results supporting the landing direction influences, our outcomes did not support hypothesis regarding the gaze direction effect. Although a significant effect of the motor task on landing stabilisation and muscle activity patterns was observed, the effects of gaze direction, both independently and in combination with the motor task, were inconclusive.

Regardless of the time interval used for computation, gaze directed downward was associated with a deterioration in sagittal dynamic stability index but an improvement in transverse dynamic stability index. Significant interactions between motor task and gaze direction effects were only observed for 3s computational time interval in the sagittal plane, suggesting that gazing straight ahead may be more advantageous during landing from SF, whereas gazing downward may be more beneficial during landing from SB. Previous studies have shown that gaze direction influences head (Otsuka et al. 2016; Fang et al. 2015) and trunk orientation (Charbonneau et al. 2024). Coordination between eye‐head movements has been shown to be proportional to the amplitude of eye and head movements. The study by Santello et al. (2001) showed that the head moves little for gaze shifts to a target near fixation but moves with the eyes for gaze shifts to a target far from fixation. These changes can significantly affect the kinematics of the trunk during landing depending on the direction of gaze. They can also result in adjustments to muscle activity, thereby influencing landing stabilisation (McNitt‐Gray et al. 2001). However, our study was focused on lower body kinematics and muscle activity. Activity patterns of the lower limb muscle groups were not influenced by gaze direction further supporting the idea about movement changes restricted to upper body, instead of chaining to the lower parts. Furthermore, the changes in stabilisation related to the combination of motor task and gaze direction were only observed for longer computation time interval of 3 s, making the results relevant only for the final elements. In terms of performing elements during routines in artistic gymnastics, the 1‐s interval is more important from a practical point of view due to the risk of possible point deduction for delayed performance (Fédération Internationale De Gymnastique 2022).

Interestingly, the effect of motor tasks was significant for dynamic stability index across all directions and planes consistently for both time intervals used for computation with addition to specific times to stabilisation. With the exception of sagittal plane of movement, the highest dynamic stability index values were generally observed during landing from AS. The greater stabilisation demands after AS can be attributed to the increased height achieved during the final fly phase in AS. Several studies confirmed changes in lower limb kinematics or lower limb muscle activity patterns related to the drop height (see reviews by Santello 2005). Gymnasts can reach heights of up to 2 m during acrobatic manoeuvres (Giottes et al., 2011), which considerably elevates the landing difficulty compared to other performed tasks. The lowest observed values of dynamic stability index during DL and lowest muscle activity before landing during DL, except from activity pattern of *m*. gastrocnemius medialis, further support this explanation. Significant differences found for time to stabilisation did not follow these trends and generally showed the highest times for DL. However, the absolute differences between conditions were marginally low. Another finding possibly associated with task height is the observation of higher muscle activity during the 300 to 150 ms period preceding landing from a SB compared to other motor tasks for the *m*. tibialis anterior and *m*. rectus femoris. Although the gymnasts performed SB from an elevated surface, we assume that their centre of mass reached lower height compared to AS and SF. This could imply the need for a faster rotation during the somersault to ensure a safe landing. Faster rotation can be achieved by increasing body tuck position, which may explain the higher activity of the *m*. rectus femoris, responsible for hip flexion. Additionally, the increased activity of the *m*. tibialis anterior indicates early dorsiflexion, again in preparation for a safe landing from a lower height. In contrast, landing after forward somersaults resulted in increased muscle activity during the 300 to 150 ms period preceding landing in the *m*. peroneus longus, *m*. gastrocnemius medialis and *m*. biceps femoris. This is because rotation in forward somersaults is mainly controlled by knee flexion due to the hands gripping the proximal part of the tibia from the ventral side. In contrast, during backward somersaults, gymnasts use a grip on the dorsal side of the distal femur, which accelerates rotation mainly through the aforementioned flexion of the hips.

In sagittal plane of movement, the highest values of the dynamic stability index and time to stabilisation were consistently observed during the landing from SF. Our hypothesis regarding the gaze direction effect was formed based on the studies showing that gaze fixation point influences prelanding body kinematics (Heinen et al. 2012; Natrup et al. 2020). However, based on our results, gaze direction does not seem to be playing an important role for lower body stabilisation during landing execution. Instead, the height and direction of landing seem more important. It has been shown that anticipation plays a crucial role for the landing execution with influencing the body kinetics and kinematics during landing and muscle preactivation before contact with the ground (Zhang et al. 2019). The ability to anticipate and respond appropriately plays a crucial role not only in ensuring safety but also in improving the quality of landings (Loffing and Cañal‐Bruland 2017). Accordingly, *m*. peroneus longus, *m*. gastrocnemius medialis and *m*. biceps femoris all showed higher prelanding activity during SF compared to other motor tasks in our study. Therefore, the key role might not be with the gaze fixation point itself, but the overall information perceived from visual system since peripheral vision is more important for the whole scene recognition than the central vision (Larson and Loschky 2009). Therefore, the difficulty of landing forward may be influenced by minimal overall visual control of the ground with gymnasts controlling rotation blindly, relying solely on spatial and kinesthetic perception (Takei et al. 2003, 2007). In contrast, during landing backward, gymnasts can observe the ground before landing and adjust their technique accordingly based on visual feedback (Natrup et al. 2020). This difference makes the stabilisation demands during a SF predictably higher; therefore, muscle activity intensifies, thereby enhancing the neuromuscular system's preparedness for the imminent load (Liebermann and Goodman 2006). Neuromuscular readiness is crucial for achieving optimal muscle stiffness and ensuring adequate braking movement to protect the joints from injury and maintain the necessary stability (Mrdakovic et al. 2008). Coaches could integrate exercises that simulate high‐stabilisation demands, such as landing drills from various heights and rotations, to improve muscle preactivation and neuromuscular readiness. Moreover, incorporate training methods that enhance gymnasts' anticipatory abilities such as reaction drills and exercises that improve spatial awareness, rather than emphasising gaze fixation alone.

Our study has several limitations. First, the limited sample size was due to the complexity of the testing, which excluded lower‐level gymnasts who were unable to achieve a sufficient number of valid landings. Second, the study focused exclusively on lower limb muscle activity patterns and lower back stabilisation. Incorporating trunk muscle activity patterns could provide more comprehensive insights and would be a valuable direction for future research. A third limitation of the study is the inability to achieve precise control of gaze direction as it was monitored visually by the researchers rather than using an electronic device such as eye‐tracking technology. The authors acknowledge that eye‐tracking technology has been employed in previous studies, and therefore, the absence of an objective measure of eye behaviour in this study represents a notable limitation. However, the participating gymnasts were unaccustomed to wearing glasses or similar equipment, which could have impaired their concentration and posed a potential safety risk.

## 5 Conclusions

This study investigated the effects of gaze direction instruction on postural stabilisation and muscle activity during landings following four motor tasks in gymnastics. Although motor task significantly influenced muscle activity patterns and stabilisation outcomes, gaze direction instruction had minimal impact, both independently and in combination with the motor task. Higher stabilisation demands were observed during landing after acrobatic series and forward somersault, likely due to greater landing heights and rotational dynamics. Gaze‐directed changes were primarily confined to sagittal stabilisation in extended time intervals, indicating limited practical implications for artistic gymnastics performance. These results highlight the importance of anticipatory muscle preactivation and visual‐spatial perception over central gaze fixation for correct landings. Future investigations focusing on upper body kinematics and peripheral visual strategies with using electronic gaze tracking devices that could provide a more comprehensive understanding of stabilisation mechanisms.

## Funding

This work was supported by Palacký University Olomouc [IGA_FTK_2024_007].

## Ethics Statement

The testing was conducted following approval from the Ethics Committee of the Faculty of Physical Culture, Palacký University Olomouc under reference number 114/2023.

## Consent

All participants and their legal guardians were informed about the study's purpose, the measurement procedures, potential risks, injury prevention strategies, data handling methods and the option to withdraw from the research at any time. Participants over the age of 18, or the legal guardians of younger participants, signed an informed consent form.

## Conflicts of Interest

The authors declare no conflicts of interest.

## Supporting information


Supporting Information S1

## Data Availability

Full data set is publicly available on https://doi.org/10.5281/zenodo.18152675 and in the supplementary file.
